# Understanding the diversity of genetic outcomes from CRISPR-Cas generated homology-directed repair

**DOI:** 10.1038/s42003-019-0705-y

**Published:** 2019-12-06

**Authors:** Brett M. Sansbury, Amanda M. Hewes, Eric B. Kmiec

**Affiliations:** 1 Gene Editing Institute, Helen F. Graham Cancer Center & Research Institute, Christiana Care Health System, Newark, DE USA; 2 Department of Medical and Molecular Sciences, University of Delaware, Newark, DE USA

**Keywords:** DNA damage and repair, CRISPR-Cas systems, CRISPR-Cas9 genome editing, CRISPR-Cas systems

## Abstract

As CRISPR-Cas systems advance toward clinical application, it is essential to identify all the outcomes of gene-editing activity in human cells. Reports highlighting the remarkable success of homology-directed repair (HDR) in the treatment of inherited diseases may inadvertently underreport the collateral activity of this remarkable technology. We are utilizing an in vitro gene-editing system in which a CRISPR-Cas complex provides the double-stranded cleavage and a mammalian cell-free extract provides the enzymatic activity to promote non-homologous end joining, micro-homology mediated end joining, and homology-directed repair. Here, we detail the broad spectrum of gene-editing reaction outcomes utilizing Cas9 and Cas12a in combination with single-stranded donor templates of the sense and nonsense polarity. This system offers the opportunity to see the range of outcomes of gene-editing reactions in an unbiased fashion, detailing the distribution of DNA repair outcomes as a function of a set of genetic tools.

## Abstract (web summary)

Brett Sansbury et al. show that a CRISPR-Cas complex that provides double-stranded cleavage combined with a mammalian cell-free extract can enhance non-homologous end joining and homology-directed repair. They use the system to show the range of genetic outcomes of gene-editing.

## Introduction

The evolution of clustered regularly interspersed palindromic repeats (CRISPR) and CRISPR-associated Cas nucleases has given researchers a novel tool with which to disable malfunctioning genes or to correct single-base mutations or small mutagenic deletions that are responsible for devastating disorders. CRISPR-Cas originated as a form of bacterial adaptive immunity^1^ and has been translated for use in human cells by innovative modifications^2,3^. The simplicity of design coupled with the efficiency of activity enables a broad spectrum of clinical development and introduction into patient populations has already begun^3,4^.

In mammalian cells, DNA cleavage activates DNA damage response pathways^5,6^, including circuitry that can lead to end modification prior to re-ligation. It is likely that non-homologous end joining (NHEJ) is the primary pathway that the cell employs to rejoin the broken template or broken chromosome^7–9^. NHEJ is generally regarded as error-prone because insertions and deletions (indels) are often generated during the process of repair^10,11^. There is evidence that the degree of NHEJ activities, such as end resection and processing, are much higher on transcriptionally active strands as compared to inactive strands^12^. This outcome might be due to the accessibility of nucleases to the transcriptionally active strand during the repair process. NHEJ and other repair pathways, including micro-homology mediated end joining (MMEJ) and/or single-strand annealing (SSA)^13,14^ likely account for genetic disruption and gene knockout.

When an appropriate donor DNA template is present in a gene-editing reaction, genetic information can be transferred from that template to its target following CRISPR-Cas cleavage. This process has been described as homology-directed repair (HDR), a broad-based terminology that could encompass several pathways^15^. These pathways share a common molecular theme where complementary DNA strands of opposite polarity are aligned in homologous register and the D-loop/joint molecular structure is resolved through replication and repair^16–18^. Most of our understanding of the DNA pairing reaction comes from fundamental studies of homologous recombination (HR) in fission yeast, budding yeast and fungus^19,20^. HDR is primarily active during meiosis to ensure proper segregation of chromosomes in the first-round of meiotic division^21^. However, somatic cells utilize various forms of HDR in order to preserve genetic integrity, assist in the repair of double-stranded breaks and help reseal single-strand DNA gaps. HDR reactions may include successful and precise insertion of a short stretch of DNA, which might be important in the repair of a mutagenic deletion within the chromosome.

In CRISPR-directed gene-editing reactions, both error-prone NHEJ and homology-directed repair compete for and act upon the same cleaved DNA site. Thus, as CRISPR-Cas systems advance toward clinical application, it becomes increasingly important to ensure that researchers and physicians can obtain all outcomes of a specific gene-editing reaction. These data will enable a more educated choice surrounding the types and amounts of genetic engineering tools to employ for the treatment of a genetic disease. For example, while high levels of single-point mutation repair have been widely reported, the accompanying secondary genetic outcomes have not been completely described. Thus, a global view of gene-editing activity is likely to be foundational as to whether to move forward with clinical application or not.

We have taken a decidedly reductionist approach to this problem by studying the mechanism of CRISPR-directed gene-editing in a system that employs a mammalian cell-free extract to drive the gene-editing reaction^22,23^. In this system, we can carefully control the level of reaction components and as a result, we can catalog the distribution of insertions, deletions and duplications as a function of each set of genetic tools. Here, we extend and utilize this cell-free system for the evaluation of homology-directed repair. We compare the applicability and efficiency of Cas9 and Cas12a ribonucleoprotein (RNP) complexes to execute double-stranded breaks in the target DNA in combination with single-stranded DNA donor templates of sense and nonsense polarities, to generate precisely modified DNA of target molecules. We designed our DNA donor templates to contain flanking regions containing arms of sequence homology to the target sequence and included an eight-base pair *Not*I restriction enzyme site, similar to that presented by Schumann et al.^24^. The successful outcome of precise HDR activity is measured by the insertion of the intact restriction enzyme sequence in proper orientation. Importantly, we identify and catalog the population of products associated with non-homologous end joining, precise homology-directed repair and error-prone repair. The genetic profiles of modified target molecules vary greatly in Cas9- and Cas12a-catalyzed reactions and the levels of precise homology-directed repair appear to be highly dependent on the Cas protein used and the polarity of the donor DNA template. Our system could serve as a screening tool, providing guidance for the selection of the most effective and least disruptive genetic tools for clinical protocols.

## Results

Our studies have been facilitated by an in vitro gene-editing system in which a CRISPR-Cas complex provides the double-stranded cleavage and a mammalian cell-free extract provides the enzymatic activity to promote NHEJ, MMEJ, and HDR in the presence of a donor DNA template. This in vitro system can promote DNA addition with either single- or double-stranded donor templates^23^. Our previous results indicate that small-fragment insertion and target site deletion are both catalyzed by the cell-free extract in a single-reaction mixture. Here, we exploit the same strategy to explore the reaction mechanics of homology-directed repair using a single-stranded DNA donor with two arms of homology to the target site.

**Fig. 1:**
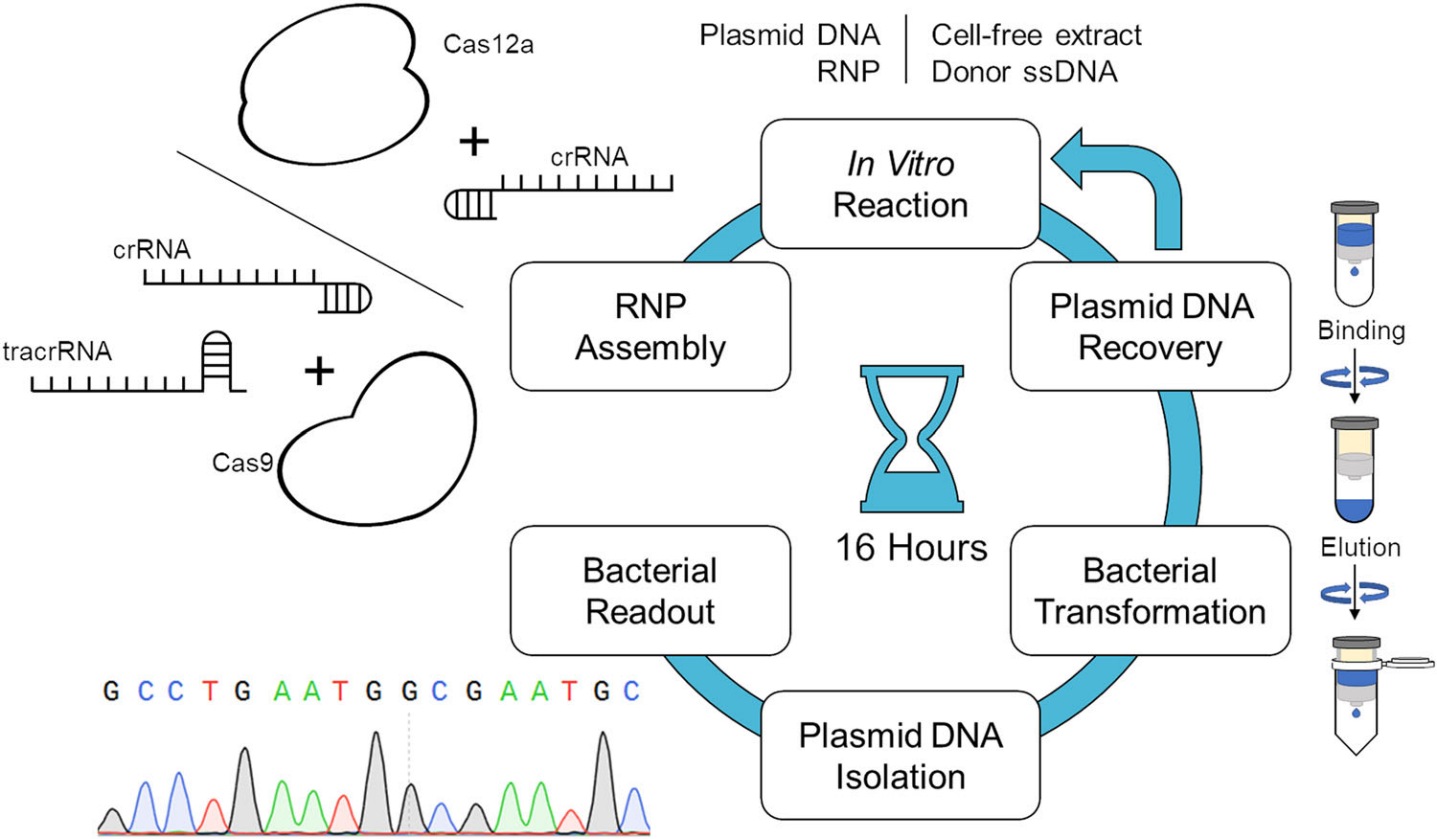
In vitro reaction experimental overview. A Cas12a or Cas9 RNP is combined with plasmid DNA, a cell-free extract and a donor DNA template in an in vitro reaction mixture. The DNA is then recovered from the in vitro reaction and transformed into competent *E. coli*. cells. Bacterial colonies are selected individually for sequencing and mutational analysis. The entirety of this process takes ~16 h to complete.

The system is outlined in Fig. 1 and the overall reaction takes ~16 h to complete. The CRISPR–Cas complex is created through RNP assembly, which initiates DNA cleavage of the target site; the cell-free extract and donor DNA are subsequently added, and the gene-editing reaction is completed. Plasmid DNA is recovered, transformed into bacteria and re-isolated using a bacterial readout. The products of the reaction are then analyzed by DNA sequencing. For each reaction, we isolated over 30 colonies so that a true representation of the gene-editing products is presented. We define HDR as the precise insertion of the eight-base pair *Not*I restriction enzyme site, as compared to error-prone repair, which includes partial insertion, deletion or a combination of both, driven by NHEJ and/or MMEJ.

**Fig. 2:**
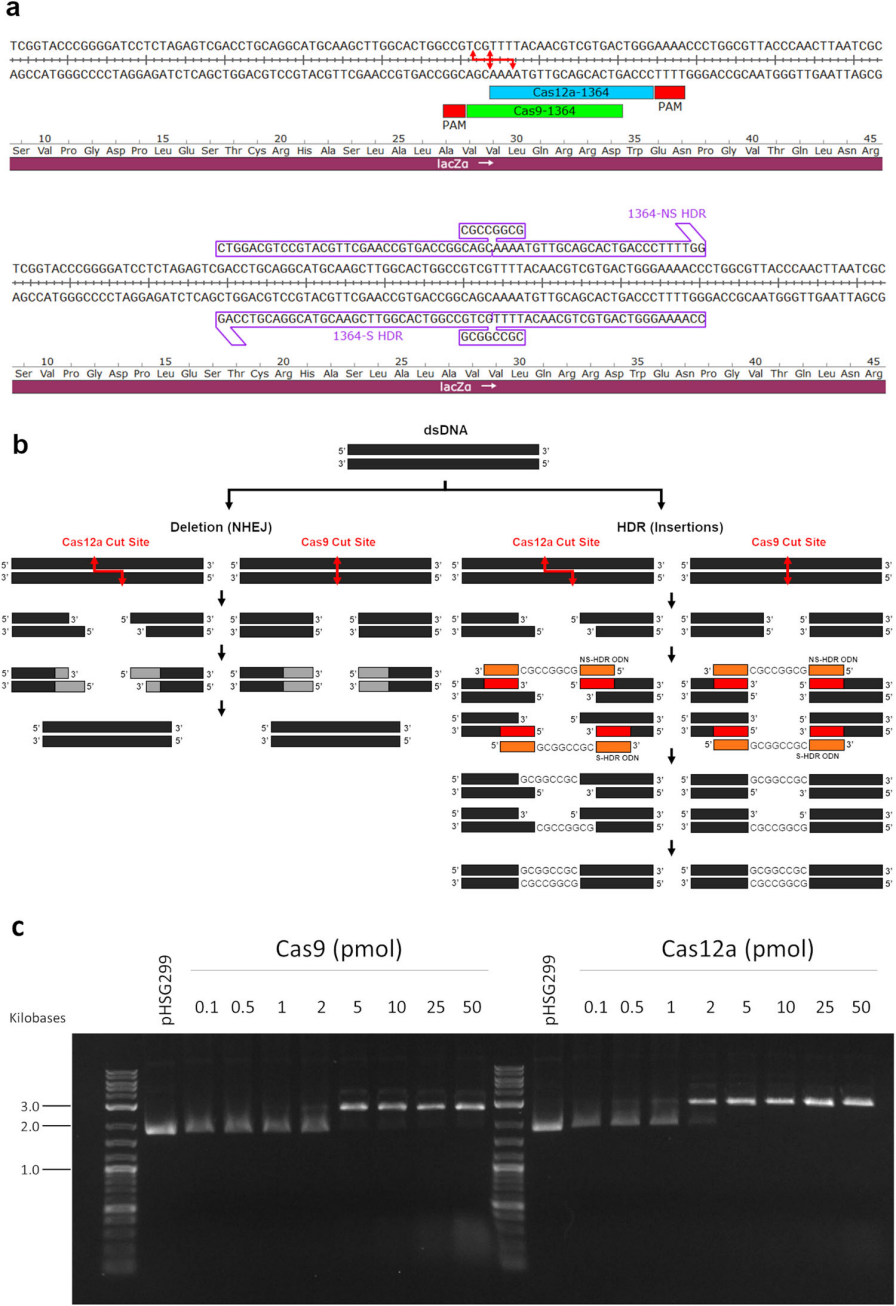
In vitro targeting and potential outcomes. **a** The target sites for Cas9 and Cas12a are shown along the *lacZ* gene with corresponding 1364-S and 1364-NS HDR templates. **b** An illustration is shown of the potential outcomes of in vitro reaction products. **c** An agarose gel is shown to display the cleavage of pHSG299 plasmid DNA by Cas9 and Cas12a at increasing pmol amounts in the in vitro system.

We have previously demonstrated that this in vitro system is fully functional on a variety of DNA targets, including eukaryotic genes^23^. For this study, we chose to target a plasmid containing the beta galactosidase gene, *lacZ*, as it provides simplified visualization of gene-editing activity through an alteration in colony color from blue to white, the typical ratio of which can be seen in Supplementary Fig. 1. We have chosen a position within the *lacZ* gene where genetic alterations of the coding sequence will disrupt the coding region to adversely affect protein function **(**Fig. 2a). To address the possibility that colonies exhibiting a blue color contain indels, likely in multiples of three, we sequenced the targeted plasmids of 118 blue colonies (Supplementary Fig. 2a–d and Supplementary Table 1). All sequences were found to contain unaltered sequence; so that to the best of our knowledge the blue/white distinction reflects either wild type (blue) or genetically modified (white) gene-editing products. This observation, of course, does not exclude the possibility that modified plasmid DNA could be harbored within random blue colonies, but we suggest that this would be a rare event. Two single-stranded DNA oligonucleotides that serve as the donor fragments are illustrated above their respective regions of complementarity in Fig. 2a; we have termed them 1364-NS to designate a donor fragment that is complementary to the sense strand and 1364-S, which is complementary to the nonsense strand. Both donor fragments are 70 bases in length and contain an eight-base *Not*I restriction enzyme site in the center flanked by two regions of homology; 35 bases upstream from the cut site and 27 bases downstream. There is no native *Not*I site within the targeted plasmid, thus HDR activity can also be screened by *Not*I digestion and gel electrophoresis (Supplementary Fig. 3).

CRISPR-Cas9 creates a blunt ended cut whereas the CRISPR-Cas12a complex generates staggered five base, 5′ overhangs^25,26^. Multiple pathways of repair are active simultaneously on a cleaved DNA molecule. In the absence of donor DNA, some form of NHEJ and/or MMEJ^27^ reconnects the linear plasmid template; in some cases, termini resection generates a deletion at the target site. In the presence of donor DNA, some form of homology-directed repair takes place potentially leading to precise or error-prone repair. Donor DNA serves as a template for replication and the complementary sequence is incorporated into the target molecule (Fig. 2b).

In Fig. 2c, we provide an analysis of the DNA cleavage activity of Cas9 and Cas12a complexes with increasing pmol amounts. Both nucleases display high activity and thus the target plasmid enters the repair phase of the reaction as fully linearized molecules. The incubation time for the gene-editing reaction was fixed at 15 min based on prior studies^22^. We did however extend the incubation time to confirm that the array of repair products generated would remain consistent; our data suggest that no notable changes in the distribution of products is observed after an extension to 90 min of reaction time (see Supplemental Fig. 4a–d).

### CRISPR-Cas9 and 1364-S template

**Fig. 3:**
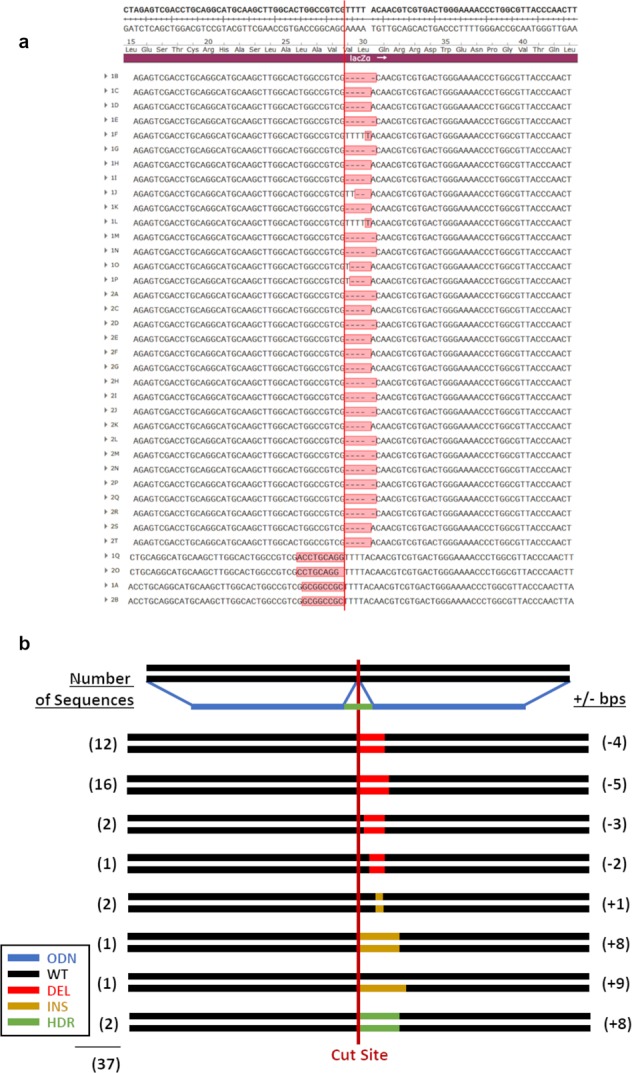
Cas9 and 1364-S template. **a** Sequences collected from reactions utilizing a Cas9 nuclease and 1364-S template are shown. **b** Illustration of the frequencies of all recorded outcomes are shown.

Our first combination included a CRISPR-Cas9 complex and a single-stranded donor template complementary to the nonsense strand, 1364-S. In Fig. 3a we display a snapshot of 87 bases of target DNA sequence with the localized position of the CRISPR-Cas9 cleavage site and list the genetic outcomes generated from this reaction. All the sequences presented have been selected from white colonies where gene-editing activity has taken place. For convenience, we provide Fig. 3b as an illustrative representation of the types of genetic alterations found within the population generated from this pair of gene-editing tools. From the 37 colonies sequenced, only two were found to contain a precisely inserted *Not*I site, the indication of precise HDR activity. The remaining colonies within the population exhibited small indels ranging from −5 bases to + 1 base. Interestingly, 28 out of 37 samples were approximately equal in distribution between deletions of 4 and 5 bases. Indel formation appears directional, with indels downstream from the cut site. The results of this experiment indicate that ~5.4% of the product molecules exhibit precise repair and NHEJ products predominate CRISPR-Cas9 reaction outcomes.

Analyses of the underlying DNA sequence generated from the precise HDR reaction confirms that the donor DNA molecule is acting as a replication template, as the inserted sequence within the targeted plasmid is the complement of the donor DNA template. Our data suggest that the insertion of complementary DNA occurs through replication extending at least four bases upstream and eight bases downstream, but does not include the entire donor strand. This mechanism of repair for CRISPR-Cas9 gene-editing was proposed by Rivera-Torres et al.^28^ after analyzing a wide number of gene-editing products in mammalian cells. We do not mean to suggest that this is the only mechanism and in fact direct integration of the donor strand could occur if polarity of the donor and the target strand are such that they permit direct incorporation.

### CRISPR-Cas9 and 1364-NS template

**Fig. 4:**
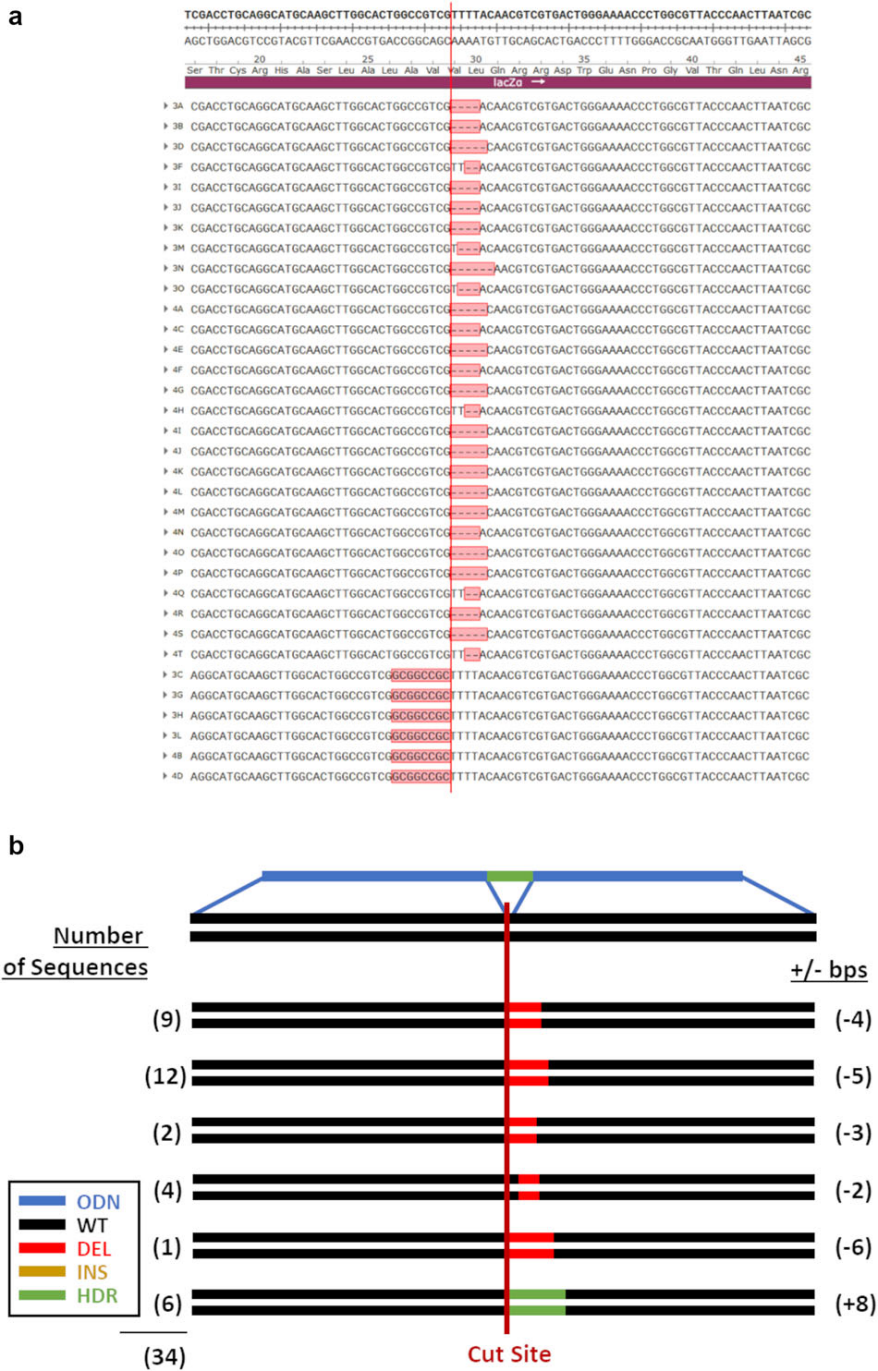
Cas9 and 1364-NS template. **a** Sequences collected from reactions utilizing a Cas9 nuclease and 1364-NS template are shown. **b** Illustration of the frequencies of all recorded outcomes are shown.

Next, gene-editing reactions containing CRISPR-Cas9 and a single-stranded donor template complementary to the sense strand were tested. From this reaction, 34 bacterial colonies were analyzed. As seen in Fig. 4a, indel formation dominated the population of isolated molecules again. Figure 4b provides an illustrative representation of the product molecules. Six of the 34 colonies harbor precise repair; representing 17.6% of the overall population. Once again, small indels of −4 and −5 base deletions were found to be the most prevalent and localized downstream from the intended cut site. Our results show that HDR activity is elevated threefold when the 1364-NS donor template is coupled to CRISPR-Cas9. A preferential polarity of the donor template relative to the target site has been noted routinely in CRISPR-directed gene-editing reactions in some^2,29^, but not all studies.

### CRISPR-Cas12a and 1364-S template

**Fig. 5:**
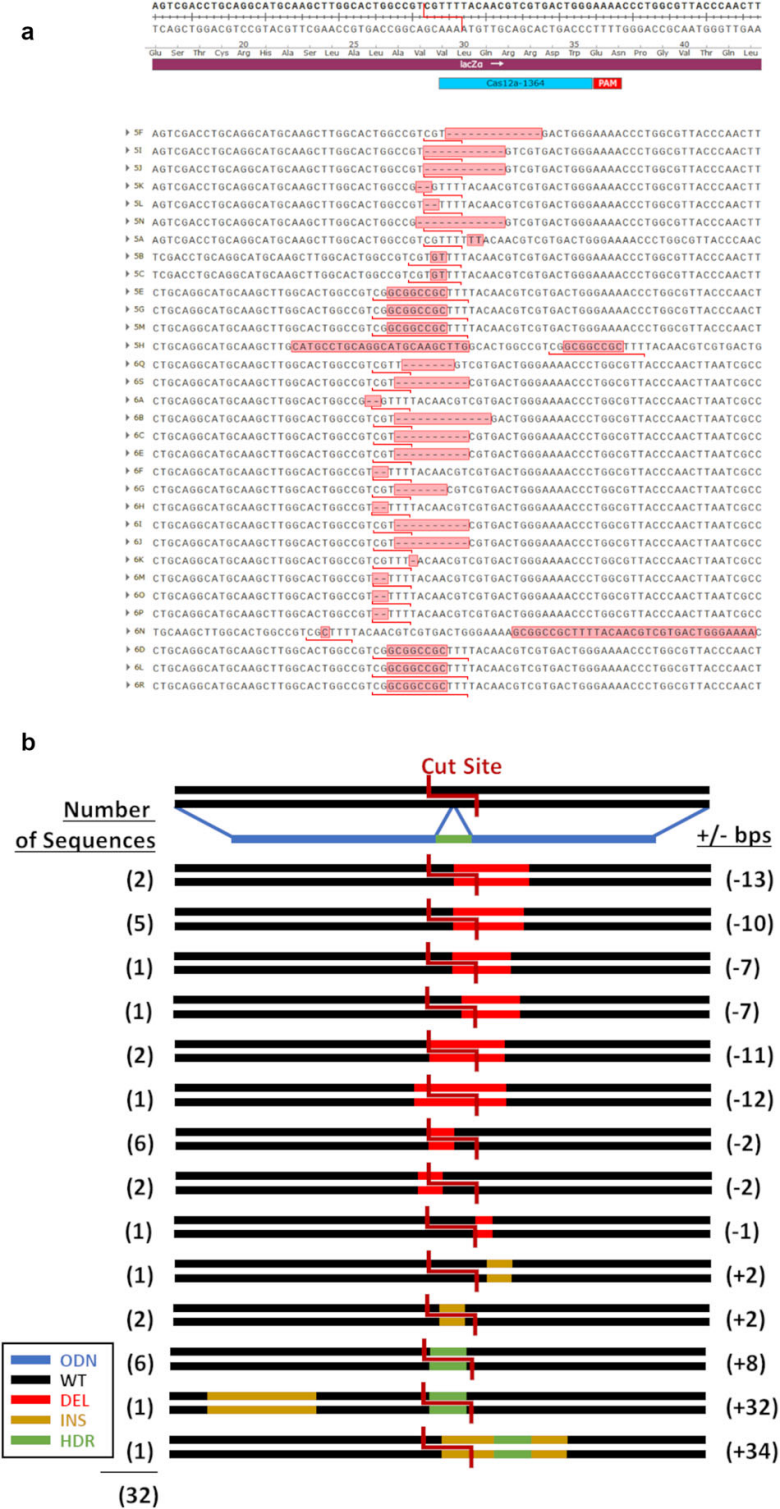
Cas12a and 1364-S template. **a** Sequences collected from reactions utilizing a Cas12a nuclease and 1364-S template are shown. **b** Illustration of the frequencies of all recorded outcomes are shown.

In the following series of experiments, we utilize Cas12a in place of Cas9 with the 1364-S donor template and the products are outlined in Fig. 5a, b. The complexity of gene-editing outcomes resulting from these conditions is broader with more uniquely mutated products. While most products harbored deletions ranging from −7 to −13 bases, we now observed both small and large insertions ranging from + 2 to + 26; insertions and deletions of this size were not observed in either reaction catalyzed by CRISPR-Cas9 (Figs. 3a, b and 4a, b). Six of the 32 colonies analyzed contained genetically modified templates with precise repair, thus specific HDR activity was found in 18.8% of the samples. It is important to note that this 18.8% compares favorably to the 5.4% precise HDR generated in Cas9 reactions using the 1364-S donor template.

### Cas12a and 1364-NS template

**Fig. 6:**
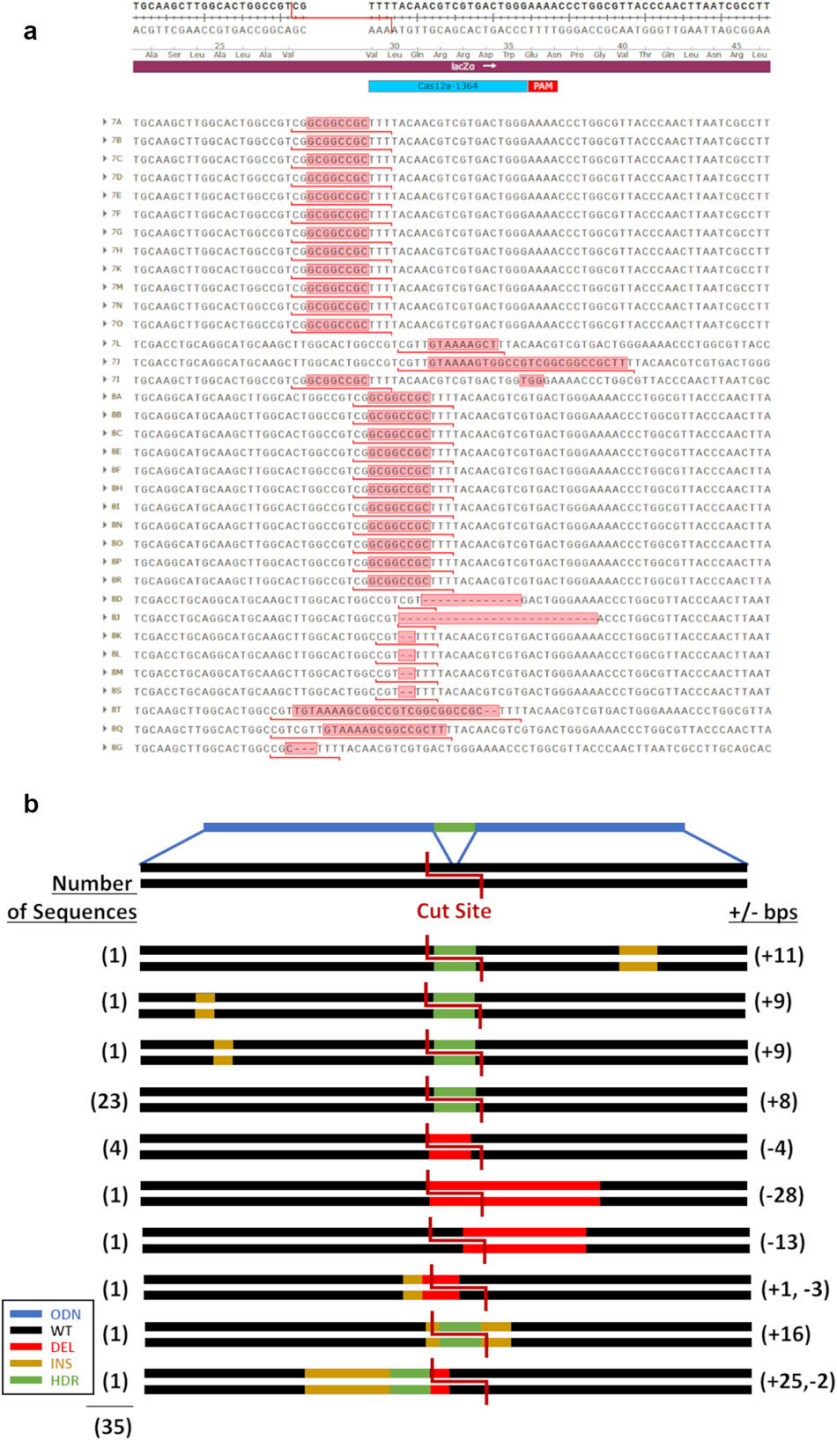
Cas12a and 1364-NS template. **a** Sequences collected from reactions utilizing a Cas12a nuclease and 1364-NS template are shown. **b** Illustration of the frequencies of all recorded outcomes are shown.

The final combination of genetic tools included Cas12a and the 1364-NS donor template. Under these conditions, 34 colonies were analyzed, and the results are presented in Fig. 6a, b. Homology-directed repair activity under these conditions is seen to be highly enhanced, with precise repair found in over 65% of the colonies analyzed. The remaining products showed a wide range of indels with deletions ranging from −4 to −28 bases and insertions from +1 to +17 bases. Not only is this the highest level of homology-directed repair observed in our study, this combination also generates the widest array of error-prone genetic modifications.

A summation of the HDR activity among all four combinations described above are shown in Table 1. From these data, we conclude the combination of CRISPR-Cas12a and the 1364-NS template exhibit the highest degree of precise HDR driven by gene-editing activity within this system. Fisher’s exact test was used to evaluate the two-tailed *P-*value for each combination described above, we provide statistical analysis using 2 × 2 contingency tables in Supplementary Table 2. After comparing the outcomes of each, Cas12a/NS alone exhibited extremely statistically significant differences to each of the remaining combinations, confirming our belief that the best combination for carrying out CRISPR-directed gene-editing is the CRISPR-Cas12a complex and the NS strand template.

**Table 1:** Frequencies of various reaction outcomes.

	Total	HDR	Indel
Cas9 1364-S	37	2 (5.4%)	35 (94.6%)
Cas9 1364-NS	34	6 (17.6%)	28 (82.4%)
Cas12a 1364-S	32	6 (18.8%)	26 (81.2%)
Cas12a 1364-NS	35	23 (65.7%)	12 (34.3%)

The total number of bacterial colonies selected from each of the four unique reaction conditions for individual sequencing and mutational analysis is shown. The number and frequencies of HDR and Indel events seen within each reaction condition are displayed.

**Fig. 7:**
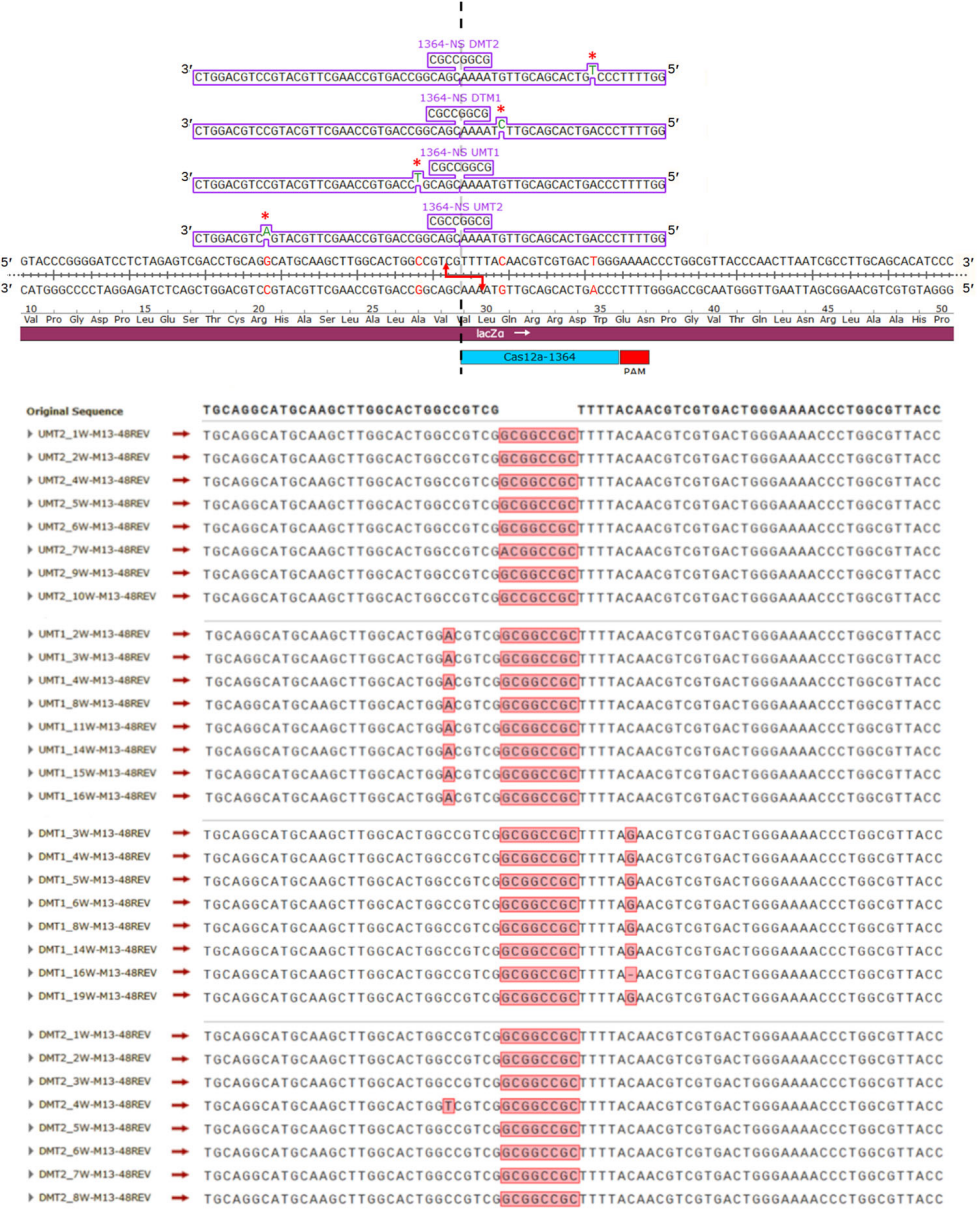
HDR template mismatch incorporation defining in vitro resection. The sequences of precise HDR reactions utilizing modified 1364-NS templates, which contain single-base mismatches 4 and 24 bases upstream and 8 and 20 bases downstream from the relative Cas12a cut site are shown.

We continued to characterize the gene-editing reaction by determining the range of distances that DNA replication fills the gap of the resection at the cleavage site. To carry out this experiment, we created four NS donor strands that have alternative nucleotides at 4 and 24 bases upstream and 8 and 20 bases downstream from the CRISPR/Cas12a cut site. As the donor DNA segment provides the template for HDR, a complementary identifiable unique base would be incorporated if replication was used to fill the gap. This approach would allow us to gain some idea of the extent of resection and replication repair that occurs during the HDR reaction, proximal to the targeted insertion site. As shown in Fig. 7, the HDR reaction was successful as previously shown and in all cases the complement of the alternative base appears four bases upstream and eight bases downstream from the segment insertion site. However, the unique complementary base is not seen at 20 bases downstream or 24 bases upstream. These data suggest that DNA resection of targeted molecule extends between 4 and 24 bases upstream and 8 and 20 bases downstream and that DNA replication activity extends within this distance. We are currently further characterizing the molecular events, including replication-dependent sequence alterations that surround the HDR reaction.

## Discussion

We have utilized an in vitro cell-free system to elucidate the mechanism of CRISPR-directed gene-editing reactions. Our long-term objective is to define the reaction parameters that surround the efficiency with which genetic knockout and genetic knock in take place. The former relies on the process of non-homologous end joining while the latter is promoted by an aggregate of pathways known as homology-directed repair^30^; it is important to note that *both* pathways are active in the same reaction when a donor DNA template is present. The cell-free system has been shown to produce deletions, insertions, and precise and error-prone repair through several recombinational repair pathways^22,23^. The insertion of small fragments is likely catalyzed by the process of MMEJ^27^ while deletions in target DNA are likely the result of resection and NHEJ^13^.

Here, we have extended the functionality of the in vitro system to homology-directed repair where the objective was to precisely insert an eight-base sequence using bilateral arms of homology^31,32^. We designed the system so that HDR could be screened by the exact insertion of a novel *Not*I restriction site and found a global view of HDR events could be visualized through DNA sequencing. We identified a single-target site in plasmid DNA that could be acted upon by different CRISPR-Cas complexes, containing with Cas9 or Cas12a. Both nucleases cleave plasmid DNA to generate linearized templates rapidly. The addition of the single-stranded donor DNA in both sense and nonsense polarities and the cell-free extract catalyzes a wide array of gene-editing products, visualized in total by genetic readout. CRISPR-Cas9 complexes successfully promote precise HDR at the designated site; CRISPR-Cas9 and the 1364-S template utilized error-prone repair with a wide array of deletions usually ranging from −2 to −6 bases and consistently appearing downstream from the cut site. In several reactions, similarities were observed between the sequences of inserted fragments, this outcome may be due to faulty usage or erroneous integration of a DNA template as reported previously by Boel et al.^33^. CRISPR-Cas9 and the 1364-NS template produced a higher level of precise HDR activity with a similar array of indels as seen previously with the experiments utilizing the S template. The 1364-NS template may be a more amenable template for the catalysis of precise HDR because it enables a more efficient DNA synthesis to take place from a preferred replication template. We next sought to characterize the resection and replication repair activity surrounding the targeted insertion site. Our data suggest that the gap filling extends between 4 and 24 bases upstream and 8 and 20 bases downstream, which helps us understand the mechanism of action. It’s important to note, however, that the resection distance and subsequent gap filling may vary between cell types^34,35^. We are investigating differences among extract sources and will report on them as soon as those detailed studies are completed.

Previous data suggests that a strand bias exists in human cell gene-editing catalyzed by programmable nucleases^22,29^. Donor template preference is recapitulated in the in vitro system with the 1364-NS template being more effective in the generation of precise HDR. Structural hindrances throughout the native genome are likely to determine partially the effectiveness of either donor templates in catalyzing gene repair through HDR. An important study by Harmsen et al.^36^ found that little or no strand bias existed in a cell-based system. Thus, the influence of strand bias should be determined on a case-by-case or target by target basis. Additionally, it is possible that symmetry or asymmetry of the donor DNA fragment itself, could influence which of the target strands is more amenable to productive gene-editing activity.

Cas12a-directed reactions utilizing the 1364-S template generated precise HDR products at a frequency similar to that observed with the Cas9/1364-NS combination. The Cas12a/1364-NS combination, however, was by far the most effective in producing precise HDR reactions, equivalent to ~65.7% of all the gene-editing outcomes analyzed. Cas12a reactions also generated more extensive, complex indel formation via error-prone repair with larger insertions and deletions. Cas12a cleavage, however, creates a more active template for NHEJ and HDR activities independent of which donor template strand is used. Hyperactivity generated by Cas12a cleavage may be accounted for by the production of DNA overhangs that can act as suitable templates for pairing proteins and cellular nucleases.

One important aspect of this in vitro system is that it affords us the opportunity to visualize the wide array of genetic modifications created through the process of CRISPR-directed gene-editing in a straightforward and simple fashion. This information is important because it provides clarity surrounding the generation of unanticipated and unintended repair products created by gene-editing tools. Such information forms the basis for determining risk-benefit decisions surrounding the effectiveness of genetic engineering tools to treat human disease.

The in vitro reaction shares significant similarities with CRISPR-directed gene-editing in human cells, including the stimulation of activity by double-stranded breaks, the dependence on DNA replication for accurate repair, the importance of a proximal cut site relative to the repair site, and the existence of a strand bias. Thus, we believe that the genetic profiles reflect the modifications in intact cells to a significant degree. We cannot however, account for the influence of cell cycle, chromatin structure and the methylation status of the target site^37–42^ on the accuracy or efficiency of gene-editing. We recognize that the categories of outcomes we report may not always appear in each experiment and certainly represents a limitation of the assay system. As we develop a comparative in vivo system that recapitulates a simple and verifiable readout as enabled by the in vitro system, several important comparisons may be made. However, transfection efficiencies and transport of the crRNA/Cas complex to the nucleus adds an inevitable variable to all cell-based systems.

The current hypothesis of how a Cas-induced double-stranded break is repaired, using single-stranded DNA as a template, focuses on the process of single-stranded template repair (SSTR)^13,18,32,36,43–46^ and confirmed by Boel et al.^33^. A similar method known as Excision and Annealing Corrective Therapy (ExACT) has been proposed to explain cell-based results where the complement of the template becomes incorporated into the target site^28^. In that study, the authors found limited categories of indels in cases where HDR did not occur, providing some support for the notion that this in vitro system can be used to predict the population of outcomes of gene-editing in intact cells. All these processes contain similar features, including initialization by a double-stranded break, resection of the 3′ end to create overhangs, pairing of the overhangs with the donor DNA template, and an extension of the 3′ strand by DNA synthesis. The products of the in vitro reaction reported herein are consistent with these models, with the exception that we utilized homology arms of 35 and 27 bases upstream and downstream from the target site, respectively^33^. We believe that this observation reflects the capacity of the 1364-NS template to anneal to the favorable 5′ overhangs created through cleavage by Cas12a. Since Cas12a leaves staggered single-stranded DNA overhangs, that molecular structure may be more amenable to resection and single-strand pairing activities. It is important to note that our experiments have been carried out using the donor DNA template that established the in vitro gene-editing system; an asymmetric molecule with a shorter 3′ end relative to the eight-base *Not*I insertion site. While asymmetric donor fragments have been used successfully in cell-based gene-editing experiments^31^, it will be important for survey the activity of symmetrical and asymmetrical donor DNA templates in the in vitro system, studies which are currently underway.

Most cell-based systems report SSTR-based genome editing at a specific locus with high variability (0.0–30%), especially when screening human cancer cell lines^32^. HDR-mediated reactions also suffer from several problems, including the fact that abortive or inaccurate, imprecise HDR is a product of CRISPR-directed gene-editing reactions^28,47–51^. We can observe error-prone events on an individual basis when either Cas9 and Cas12a and the 1364-S and 1364-NS templates, all of which are represented in each reaction profile. As most investigators, we believe that the mechanistic questions surrounding human gene-editing should be further addressed as they form the foundation for human therapy. Here, we contribute to this effort by providing a more accurate view of the multiple outcomes of CRISPR-directed gene-editing in an unbiased and highly validated fashion.

## Methods

### Cell-free extract preparation

HEK-293 cells (American Type Cell Culture, Manassas, VA) were cultured and 8 × 10^6^ cells were harvested and washed in cold hypotonic buffer (20 mM HEPES, 5 mM KCl, 1.5 mM MgCl_2_, 1 mM DTT, and 250 mM sucrose). Cells were centrifuged and re-suspended in cold hypotonic buffer without sucrose, followed by incubation on ice for 15 min before being lysed by 25 strokes of a Dounce homogenizer. Cytoplasmic fraction of enriched cell lysate was incubated on ice for 1 h and then centrifuged for 15 min at 12,000 × g, 4 °C. The supernatant was then aliquoted and stored at −80 °C. The concentration of cell-free extracts was determined using a Bradford assay.

### In vitro reaction conditions

In vitro DNA cleavage reaction mixtures consisted of 500 ng (0.014 µM) of pHSG299 (Takara Bio Company, Shiga, Japan) plasmid DNA and 10 pmol RNP in a reaction buffer (100 mM NaCl, 20 mM Tris-HCl, 10 mM MgCl_2_ and 100 µg/ml BSA) at a final volume of 20 µl. RNP complexes consisted of purified AsCas12a or SpCas9 protein (Integrated DNA Technologies, Coralville, Iowa) and site-specific crRNA (Integrated DNA Technologies, Coralville, Iowa). Each reaction was incubated for 15 min at 37 °C, after which DNA was isolated from reaction mixtures and recovered using *Select-a-Size* DNA Clean & Concentrator (Zymo Research, Irvine, CA). Secondary in vitro HDR reactions included DNA recovered from the initial cleavage reaction, 100 pmol of single-stranded donor DNA (Integrated DNA Technologies, Coralville, Iowa) 1364-S 5′-GACCTGCAGGCATGCAAGCTTGGCACTGGCCGTCGGCGGCCGCTTTTACAACGTCGTGACTGGGAAAACC-3′ or 1364-NS 5′-GGTTTTCCCAGTCACGACGTTGTAAAAGCGGCCGCCGACGGCCAGTGCCAAGCTTGCATGCCTGCAGGTC-3′ and 175 µg of cell-free extract supplemented with 400 cohesive end units of Quick T4 Ligase (New England Biolabs, Ipswich, MA) in a reaction buffer (20 mM TRIS, 15 mM MgCl_2_, 0.4 mM DTT and 1.0 mM ATP) at a final volume of 25 µl. Each reaction was then incubated for 15 min at 37 °C. Modified plasmid DNA from the final reaction mixture was then isolated and purified during spin column recovery.

### Transformation, DNA isolation, and analysis

Modified plasmid DNA recovered from in vitro reactions were transformed in 50 µl of DH5α competent *E. coli* (Thermo Fisher Scientific Wilmington, DE) via heat shock methodology, after which 150 µl of undiluted cells were plated on media containing X-gal and kanamycin and incubated overnight at 37 °C. Plasmid DNA was isolated from kanamycin resistant, white colonies via ZymoPURE Plasmid Miniprep Kit (Zymo Research, Irvine, CA). Modifications made to plasmid DNA were evaluated after DNA sequencing (GeneWiz, South Plainfield, NJ) and disruption patterns were assessed using SnapGene software.

### PCR Amplification

PCR amplification of the *lacZ* gene from selected bacterial colonies generated a 547 bp amplicon using PCR Primers (Integrated DNA Technologies, Coralville, Iowa) FWD 5′-GCTTCCGGCTCGTATGTTGTGTGG-3′ and REV 5′-GTTGGACGAGTCGGAATCGCAGA-3′. The PCR conditions involved an initial denaturation of template DNA at 94 °C for 2 min, cycle denaturation at 94 °C for 30 s, primer annealing at 60 °C for 1 min, and extension at 68 °C for 30 s for 35 cycles with a hold at 68 °C for 10 min. Each PCR contained an individually picked bacterial colony, 10 µM forward and reverse primers, PCR qualified water (Quality Biological Inc., Gaithersburg, MD) and *OneTaq* Quick-Load Master Mix (New England Biolabs, Ipswich, MA) in a total reaction volume of 50 µl. PCR products were purified using QlAquick PCR Purification Kit (Qiagen, Hilden, Germany).

### Statistics and reproducibility

Statistical analysis was done to determine the significance of precise HDR outcomes for each combination of Cas9, Cas12a nucleases, and 1364-S, 1364-NS donor templates used. For this analysis, Fisher’s exact test was used to evaluate the two-tailed *P*-value for each combination and the results of these analysis are provided in 2 × 2 contingency tables (see Supplementary Table 2).

### Reporting summary

Further information on research design is available in the Nature Research Reporting Summary linked to this article.

## Supplementary information

Supplementary Information

Reporting Summary

## Data Availability

All data generated throughout this study has been included in this article and the accompanying supplementary materials
