# Supplementary material for: Understanding the diversity of genetic outcomes from CRISPR-Cas generated homology-directed repair
Source: Commun Biol. 2019 Dec 6;2:458. doi: 10.1038/s42003-019-0705-y (PMC6898364; doi:10.1038/s42003-019-0705-y)
Supplement: Supplementary file 1 — Supplementary Information [file 42003_2019_705_MOESM1_ESM.pdf]

Supplementary Figures

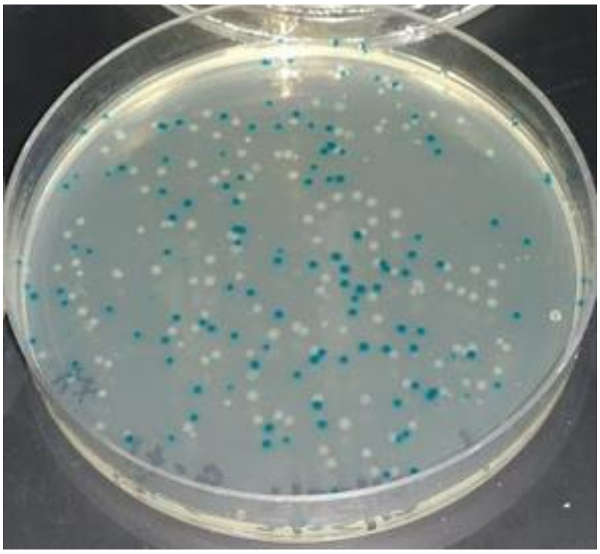

Supplementary Fig. 1: *LacZ* plasmid color change. Agar plates are shown representing a distinct color change from blue to white in bacterial colonies transformed with modified plasmids recovered from *in vitro* reactions.

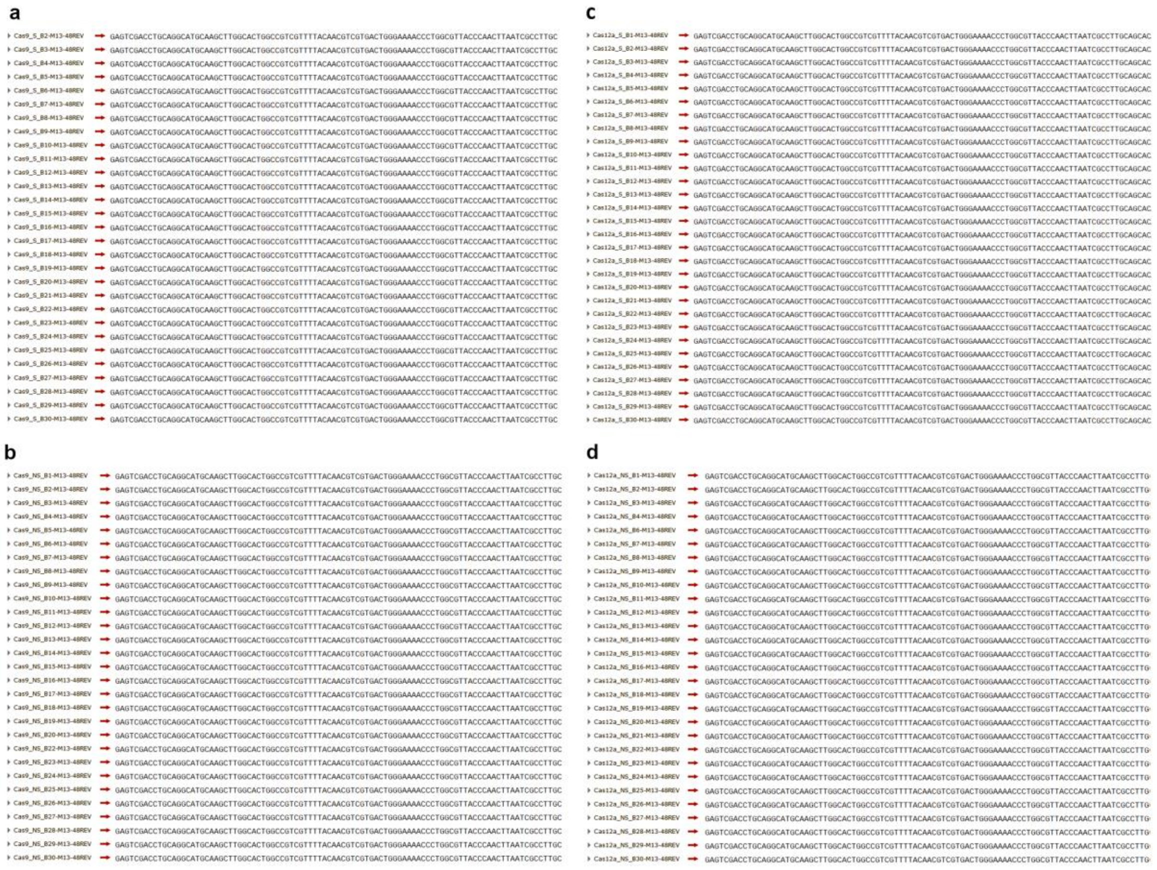

Supplementary Fig. 2: Outcomes from selected blue colonies. Outcomes are shown from selected blue bacterial colonies after *in vitro* reactions were complete under four unique conditions including **a,** Cas9 and 1364-S template. **b,** Cas9 and 1364-NS template. **c,** Cas12a and 1364-S template. **d,** Cas12a and 1364-NS template.

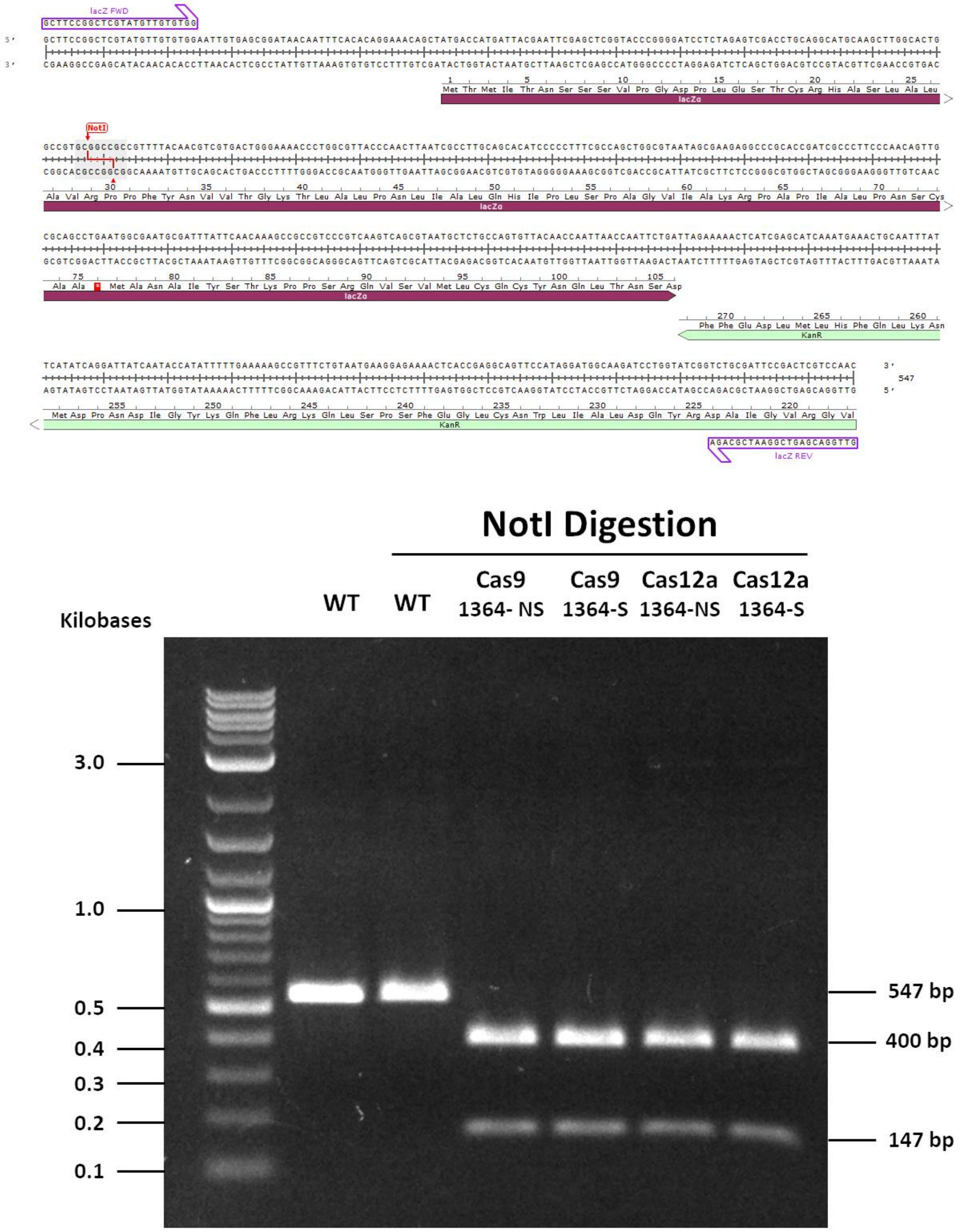

Supplementary Fig. 3: NotI Digestion of lacZ PCR amplicons generated from modified lacZ plasmids. A representation of the 547 bp *lacZ* amplicon generated from PCR of selected bacterial colonies after transformation with plasmid recovered after precise HDR has occurred. An agarose gel is shown confirming the presence of integrated NotI sites via NotI digestion of 547 bp amplicons to generate 147 bp and 400 bp fragments generated from modified plasmid DNA recovered from *in vitro* reactions via bacterial colony PCR.

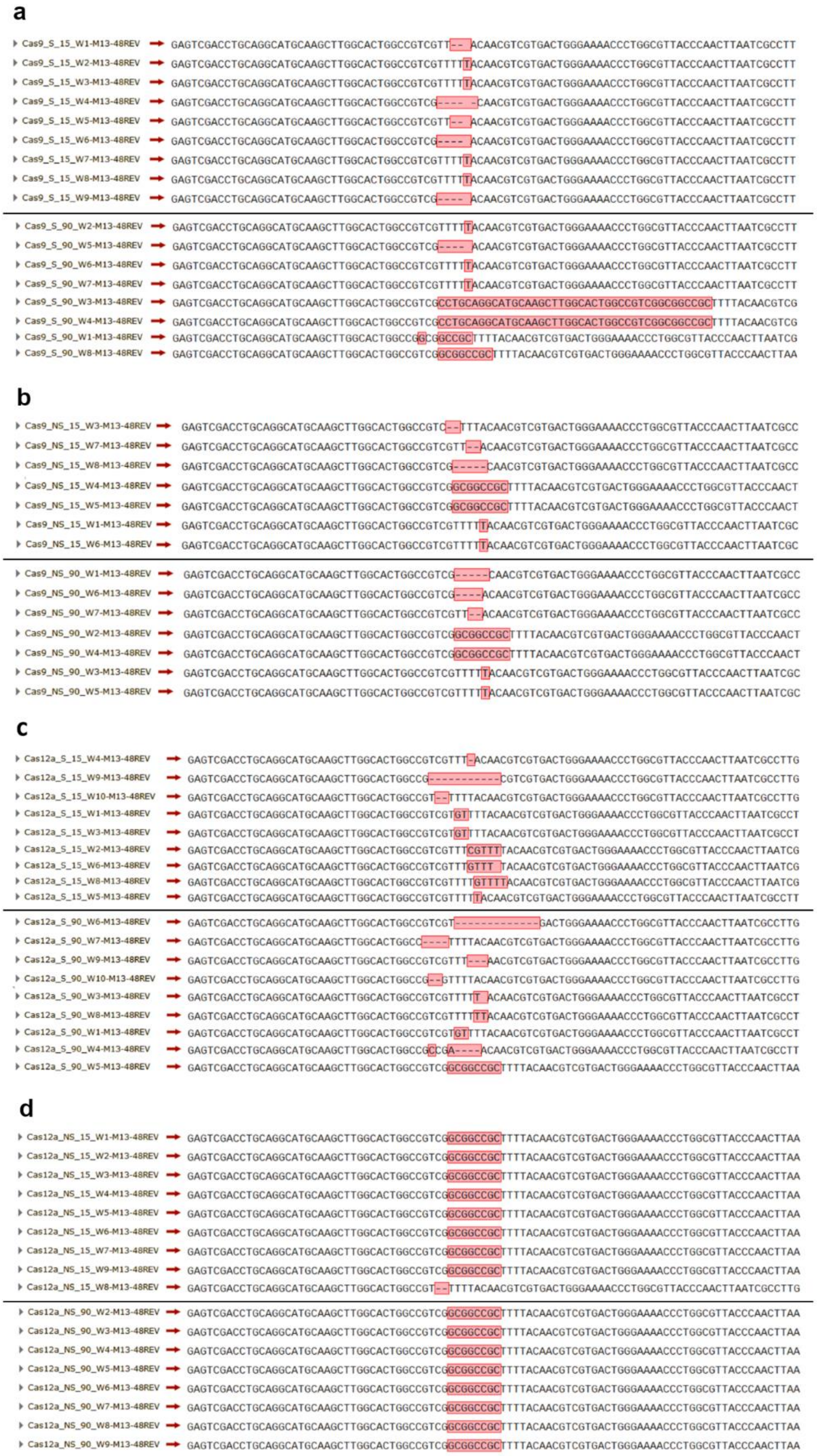

Supplementary Fig. 4: Outcomes from 15- and 90-minute reactions. Outcomes are shown from reactions that were stopped after (top) 15 and (bottom) 90 minutes which included **a,** Cas9 and 1364-S template. **b,** Cas9 and 1364-NS template. **c,** Cas12a and 1364-S template. **d,** Cas12a and 1364-NS template.

Supplementary Tables

	Total	WT	Indel
**Cas9 1364-S**	**29**	29	0
**Cas9 1364-NS**	**30**	30	0
**Cas12a 1364-S**	**30**	30	0
**Cas12a 1364-NS**	**29**	29	0

Supplementary Table 1: Summary of outcomes from blue colonies. The total number of blue bacterial colonies selected from each of the four unique reaction conditions for individual sequencing and mutational analysis is shown. The number of wild-type (WT) and Indel events seen within each reaction condition are displayed.

	HDR	Indel	Total
**Cas9/S**	2	35	**37**
**Cas12a/S**	6	26	**32**
**Total**	**8**	**61**	**69**

P = 0.1324

	HDR	Indel	Total
**Cas9/NS**	6	26	**32**
**Cas12a/S**	6	28	**34**
**Total**	**12**	**54**	**66**

P = 1.000

	HDR	Indel	Total
**Cas9/S**	2	35	**37**
**Cas9/NS**	6	28	**34**
**Total**	**8**	**63**	**71**

P = 0.1411

	HDR	Indel	Total
**Cas9/S**	2	35	**37**
**Cas12a/NS**	23	12	**35**
**Total**	**25**	**47**	**72**

P = 0.0001

	HDR	Indel	Total
**Cas9/NS**	6	28	**34**
**Cas12a/NS**	23	12	**35**
**Total**	**29**	**40**	**69**

P = 0.0001

	HDR	Indel	Total
**Cas12a/S**	6	26	**32**
**Cas12a/NS**	23	12	**35**
**Total**	**29**	**38**	**67**

P = 0.0002

Supplementary Table 2: Statistical analysis of HDR and Indel outcomes among Cas9/Cas12a and S/NS reaction combinations. Fisher's exact test was used to evaluate the two-tailed P value for each gene editing tool combination tested. Shown here are the 2x2 contingency tables generated for analysis of the outcomes between combination. Cas9/S:Cas12a/NS, Cas9/NS:Cas12a/NS, Cas12a/S:Cas12a/NS comparisons utilized n=72, 69, and 67 biologically independent samples, respectively.
